# p53 Modulation as a Therapeutic Strategy in Gastrointestinal Stromal Tumors

**DOI:** 10.1371/journal.pone.0037776

**Published:** 2012-05-25

**Authors:** Joern Henze, Thomas Mühlenberg, Susanne Simon, Florian Grabellus, Brian Rubin, Georg Taeger, Martin Schuler, Juergen Treckmann, Maria Debiec-Rychter, Takahiro Taguchi, Jonathan A. Fletcher, Sebastian Bauer

**Affiliations:** 1 Department of Medical Oncology, Sarcoma Center, West German Cancer Center, University Duisburg-Essen Medical School, Essen, Germany; 2 Department of Pathology and Neuropathology, Sarcoma Center, West German Cancer Center, University Duisburg-Essen Medical School, Essen, Germany; 3 Department of Trauma and Orthopedic Surgery, Sarcoma Center, West German Cancer Center, University Duisburg-Essen Medical School, Essen, Germany; 4 Department of Visceral and Transplant Surgery, Sarcoma Center, West German Cancer Center, University Duisburg-Essen Medical School, Essen, Germany; 5 Department of Molecular Genetics and Anatomic Pathology, Lerner Research Institute, Cleveland Clinic, Taussig Cancer Center, Cleveland, Ohio, United States of America; 6 Department of Human Genetics, University of Leuven, Leuven, Belgium; 7 Division of Human Health and Medical Science, Graduate School of Kuroshio Science, Kochi University, Nankoku, Kochi, Japan; 8 Department of Pathology, Brigham and Women's Hospital, Harvard Medical School, Boston, Massachusetts, United States of America; University of Illinois at Chicago, United States of America

## Abstract

The KIT-inhibitor imatinib mesylate (IM) has greatly improved the treatment of metastatic gastrointestinal stromal tumors (GIST). IM exhibits strong antiproliferative effects but fails to induce sufficient levels of apoptosis resulting in low pathologic complete remission rates and a high rate of secondary progression in the metastatic setting. Upregulation of p53 by MDM2 inhibitors has been shown to induce apoptosis in p53 wildtype tumors. Analyzing a series of 62 mostly untreated, localized and metastatic GIST we detected a low rate (3%) of inactivating p53 mutations, thus providing a rationale for further exploration of p53-directed therapeutic strategies. To this end, we studied nutlin-3, an inhibitor of the p53 antagonist MDM2, and RITA, a putative p53 activator, in GIST cell lines. Nutlin-3 effectively induced p53 at therapeutically relevant levels, which resulted in moderate antiproliferative effects and cell cycle arrest in p53 wildtype GIST cell lines GIST430, GIST48 and GIST48B. P53 reactivation substantially improved the apoptotic response after effective KIT inhibition with sunitinib and 17-AAG in IM-resistant cell lines. The commonly used imatinib-sensitive cell lines GIST882 and GIST-T1 were shown to harbor defective p53 and therefore failed to respond to nutlin-3 treatment. RITA induced p53 in GIST48B, followed by antiproliferative effects and a strong induction of apoptosis. Surprisingly, GIST-T1 was also highly sensitive to RITA despite lacking functional p53. This suggested a more complex, p53-independent mechanism of action for the latter compound. No antagonistic effects from p53-activating drugs were seen with any drug combination. Our data provide first evidence that modulation of the MDM2/p53 pathway may be therapeutically useful to improve the apoptotic response of KIT-inhibitory drugs in the treatment of naïve GIST, with p53 mutation status being a predictive factor of response.

## Introduction

Gastrointestinal stromal tumors (GISTs) are the most frequent mesenchymal neoplasms of the gastrointestinal tract [1] and are characterized by activating mutations of KIT or platelet-derived growth factor receptor alpha (PDGFRA) [2] [3]. Imatinib mesylate, a small molecule inhibitor of KIT and PDGFRA, yields long-lasting responses in the majority of patients [4], however, 80–90% of the patients eventually develop secondary resistance and progress with a dismal outcome. Despite major tumor shrinkage and regressive changes seen in CT scans, resection specimen contain viable tumor cells in most patients responding to imatinib [5]. While this may be attributed to pre-existing clones harboring secondary resistance mutations, these findings suggest that the inhibition of the KIT oncogenic signal alone does not sufficiently induce apoptosis.

The p53 transcription factor is an important cell cycle regulator that plays a key role in the cellular defense against neoplastic transformation [6]. Mutations of the *TP53* gene are commonly found in human tumors causing the expression of an inactive gene product. In addition, p53 inactivation can be exerted by viral oncogenic products, defects of its upstream regulator p14^ARF^ or association of p53 protein with the murine double-minute 2 (MDM2) cellular oncoprotein [7] [8]. MDM2, a major physiological antagonist of p53, can bind the p53 transactivation domain, thereby interfering with p53 transcriptional regulatory mechanisms [7] [9] [10]. MDM2 is also an E3 ubiquitin ligase that promotes p53 proteasomal degradation. MDM2 is overexpressed in some human tumors by gene amplification, thus inactivating p53 function. Reactivation of the p53 pathway through inhibition of MDM2 may result in either induction of cell cycle arrest by upregulation of p21 or induction of apoptosis even in the absence of MDM2 amplification [11]. Previous studies have demonstrated that a prerequisite for a proapoptotic effect of MDM2 inhibitors (MDM2i) in therapeutically relevant doses is the absence of inactivating p53 mutations [11]. However, nutlin-3 has also been shown to induce apoptosis in p53-null and p53-mutated cells via p73 at higher doses [12].

While almost 50% of all human tumors harbor inactivating p53 mutations, these are thought to be rarely found in GIST, thus rendering GIST to be potentially sensitive to reactivation of p53 [13].

Against this background we sought to evaluate p53 modulation as a therapeutic approach in GIST using nutlin-3 and RITA, two extensively characterized MDM2 inhibitors, both as a single agent and in combination with drugs that inhibit the KIT oncogenic pathway.

## Results

### Results of *TP53* analysis in 62 GISTs

We screened 62 GISTs regardless of p53 or p21 expression levels for *TP53* mutations to determine how often p53-function is depleted by inactivating mutations. Forty were primary and 22 were metastatic GISTs. We found a P72R-polymorphism in 37 out of the 40 primary and in 20 out of the 22 metastatic GISTs (Tab. 1). Two out of 62 tumors showed a mutation, H193R in one of the primary GISTs and H290P in one of the metastatic GIST (Tab. 2). Most patients were untreated GISTs, the metastatic GISTs harboring a *TP53* mutation had failed multiple treatment lines (imatinib, sunitinib, nilotinib and sorafenib).

**Table 1:** *TP53* sequencing results of 62 patients with primary localized (n = 40) or metastastic (n = 22) GIST: P72R-polymorphism status.

Amino Acid 72	GIST (n = 62)	%	Healthy Population* (n = 107) in %
Arg (R) homozygous	36	58	22
Pro/Arg heterozygous	21	34	65
Pro (P) wildtype	5	8	12

**Table 2:** *TP53* sequencing results of 62 patients with primary localized (n = 40) or metastastic (n = 22) GIST.

Stage/tissue	no.pts (n = 62)	Mutation	%	Polymorphism (P72R)	%
very low	5	0	0%	5	100%
low	10	0	0%	10	100%
moderate	9	1 (H193R)	11%	8	89%
high	16	0	0%	14	87%
metastatic	22	1 (H290P)	5%	20	91%

### *TP53* sequencing analysis of GIST cell lines

**Figure 1:**
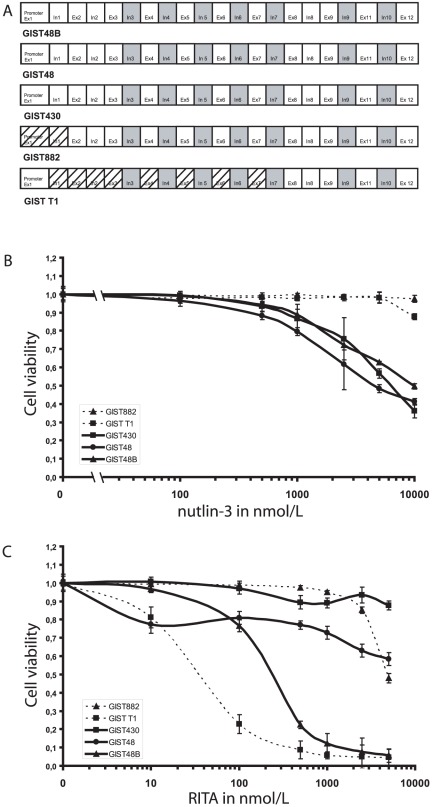
Results of TP53 sequencing and MDM2i treatment in GIST cell lines. A: *TP53* sequencing in all GIST cell lines. Dashed lines indicate that the product of the given exon/intron could not be amplified. B+C: Sulforhodamin-based cytotoxicity assays for nutlin-3 (A) and RITA (B) in IM-sensitive (GIST882, GIST-T1) and IM-resistant (GIST48, GIST48B, GIST430) cell lines. All cells were treated with the indicated concentrations and assessed after 6 days of treatment, with the data normalized to DMSO-only controls. X = 0 is DMSO-only treated. Lines, KIT-positive cell lines; dotted lines, KIT-negative cell lines, points, mean of quadruplicate cultures; bars, SD.

We first sequenced *TP53* in all GIST cell lines. No mutations were found in *TP53* (exon 1–12) in GIST430, GIST48 and GIST48B. GIST-T1 and GIST882 were wild type for exons 2–12 and 1, 8–12 but we were unable to amplify the promotor region in GIST882 and exons 2–7 in GIST-T1 suggesting homozygous deletions in these regions (Fig. 1A/Tab. 3). RNA sequencing data from several GIST cell lines supported the complete absence of p53 transcript based on approximately 7×10^7^ exonic reads in GIST882 compared to GIST48 and GIST430 (FigS1).

**Table 3:** *TP53* sequencing results of 5 GIST cell lines.

Stage/tissue	no. (n = 5)	Mutation	%	Polymorphism (P72R)	%
**Cell lines**	5	2	40%	1	20%
GIST882		suspected deletion Exon 1		1	
GIST T1		suspected deletion Exon 2–7			
GIST430		-			
GIST48B		-			
GIST48		-			

### MDM2 inhibitors exhibit differential antiproliferative effects in IM-resistant GIST cell lines

We evaluated the antiproliferative effects of nutlin-3 and RITA in five GIST cell lines by treating cells for 6 days with increasing concentrations of nutlin-3 and RITA (10 nM to 5 µM for RITA and 10 nM to 10 µM for nutlin-3) using the sulforhodamin B cytotoxicity assay. Nutlin-3 treatment resulted in antiproliferative effects in all IM-resistant cell lines (GIST430, GIST48 and GIST48B; IC50 4–10 µM), but not in IM-sensitive GIST882 and GIST-T1 (IC50>10 µM; Fig. 1B). RITA treatment caused strong antiproliferative effects in GIST-T1 and GIST48B (IC50 30 nM and 200 nM, respectively), but showed only mild effects in other cell lines (IC50>10 µM; Fig. 1C).

### Effects of MDM2i and IM on the induction of apoptosis: RITA induces apoptosis in GIST48B and GIST-T1

**Figure 2:**
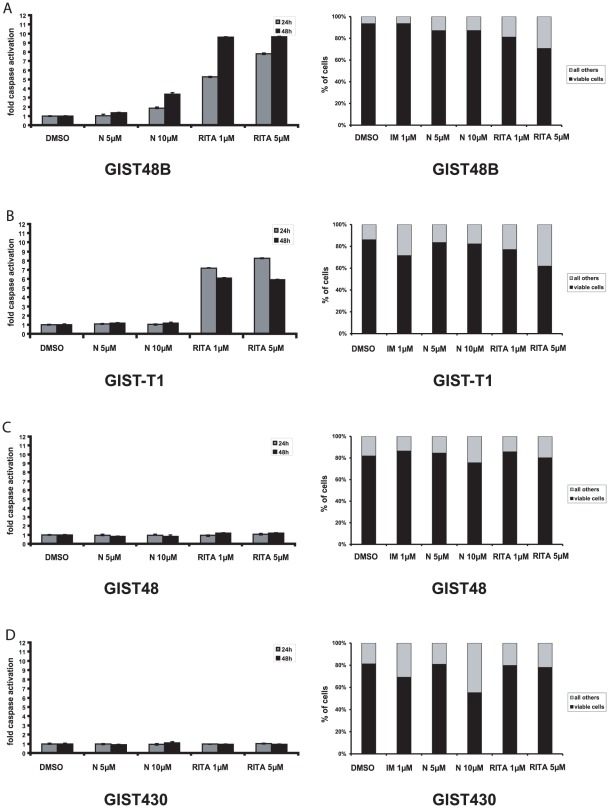
Induction of apoptosis represented by amount of activated caspases 3 and 7 (graphs on the left side) and annexin V/7-AAD based FACS assay (graphs on the right side) in GIST48B (A), GIST-T1 (B), GIST48 (C) and GIST430 (D). Indicated concentrations of the inhibitors were assayed after 24 h and 48 h of incubation. DMSO was vehicle control. Bars, mean of quadruplicate cultures with SD represent multitudes of DMSO-only values. N = nutlin-3.

We then analyzed the effects of MDM2 inhibitors on the induction of apoptosis using a luminescence based assay to measure cleaved caspases 3 and 7 as well as by determining the amount of apoptotic cells by flow cytometry using annexin V/7-AAD staining. Nutlin-3 10 µM showed a 4-fold induction of caspase 3/7 cleavage in GIST48B but not in any other GIST cell line (Fig. 2, data not shown). A mild induction of apoptotic cells as measured by flow cytometry was seen in all IM-resistant cell lines but no effects were seen in GIST882 and GIST-T1. Treatment with RITA 1 µM resulted in a 7-fold induction of cleaved caspase 3/7 in both GIST48B and GIST-T1 (Fig. 2A/B) correlating well with cytometry studies (Fig. 2A/B). GIST48 was not affected by RITA treatment (Fig. 2C/D). As expected, imatinib treatment resulted in induction of annexin V/7AAD positive cells in the IM-sensitive cell line GIST-T1, but not in the IM-resistant cell lines GIST48 and GIST48B (Fig. 2A/B/C).

### Effects of MDM2i and IM on the cell cycle: Nutlin-3 induces S-phase suppression in IM-resistant cell lines

We then performed cell cycle analyses to further evaluate the effects of nutlin-3 and RITA. In the p53 wildtype cell lines, treatment with nutlin-3 reduced cell proliferation. In GIST48 17.0% of cells were in S-Phase in the control sample, which was reduced to 6.5% after 5 µM nutlin-3 treatment. The cells in G1-Phase increased from 70.0% to 83.0% (FigS2). In GIST48B nutlin-3 treatment reduced the S-Phase from 20.5% to 5.7% and increased G1-Phase from 64.3% to 85.0% (FigS2). RITA induced apoptotic cells in GIST48B and GIST-T1 (0.9% vs. 17.5% and 4.1% vs. 17.3%; FigS2). Furthermore, in GIST48 and GIST-T1 treatment with high doses of RITA (5 µM) increased the amount of cells in S-Phase (17.0% vs. 51.6% and 12.6% vs. 35.2%) and decreased the amount of cells in G1-Phase (70.0% vs. 35.8% and 71.9% vs. 48.7%) (FigS2).

### Effects of MDM2i on p53 and p53 associated proteins (MDM2, p21, cleaved caspase 3)

**Figure 3:**
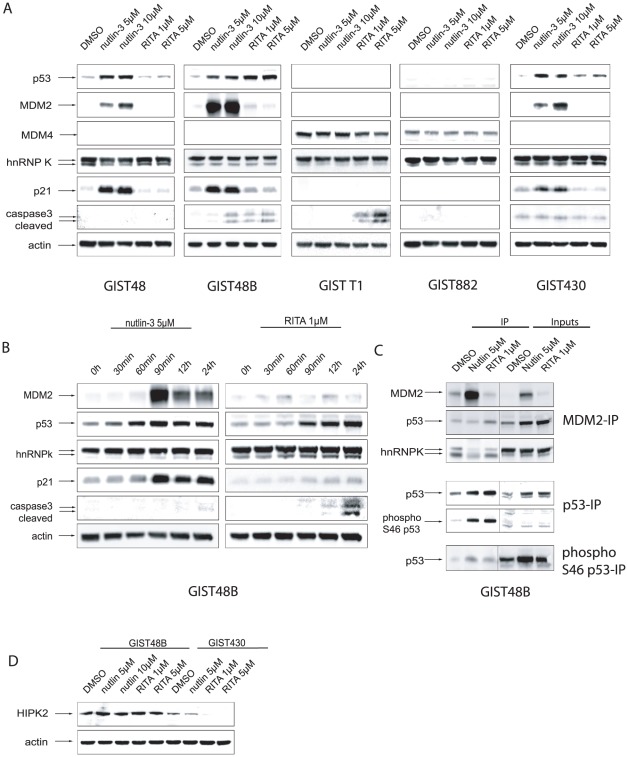
Western Blot analyses of nutlin-3 and RITA effects on p53 and p53-dependent signalling pathways. A: Western Blot analyses after 24 h of incubation with doses of nutlin-3 (5 µM and 10 µM) and RITA (1 µM and 5 µM) in GIST48, GIST48B, GIST-T1, GIST882 and GIST430. B: Time course study in GIST48B. Cells treated for indicated intervals with nutlin-3 5 µM and RITA 1 µM. C: Immunoprecipitation of MDM2, p53 and phospho-p53 from GIST48B cell lysates treated with DMSO, nutlin-3 5 µM and RITA 1 µM for 24 h hours. Staining of whole cell lysates (Inputs) for the indicated proteins. D: Western Blot analyses after 24 h of incubation with doses of nutlin-3 (5 µM and 10 µM) and RITA (15 µM) in GIST48B and GIST430.

We further investigated the effects of nutlin-3 and RITA treatment on p53 and p53-dependent signaling pathways by western blot analysis. Figure 3A displays the effects of the indicated treatment after 24 hours of incubation. Figure 3B is a time course that covers the effects of nutlin-3 treatment at different time points between 30 minutes and 24 hours. Nutlin-3 treatment (5 µM and 10 µM) strongly induced p53 expression after 60 minutes, as well as MDM2 and p21 expression after 90 minutes in IM-resistant cell lines GIST48, GIST48B and GIST430. GIST882 and GIST-T1 (Fig. 3A/B) did not express p53 at baseline or after MDM2 inhibition. In GIST48B, 10 µM treatment led to substantial caspase 3 cleavage (Fig. 3A).

In GIST48B treatment with RITA strongly induced p53 after 90 minutes and a cleavage of caspase 3 after 24 h, without affecting levels MDM2 or p21 (Fig. 3A/B). In GIST-T1 RITA treatment induced a marked cleavage of caspase 3, but no induction of MDM2 (Fig. 3A/B).

### Nutlin-3 inhibits the binding of MDM2 to p53 and hnRNP K

To test whether MDM2i act by disrupting the binding between MDM2 and p53, we performed an immunoprecipitation of MDM2. Treatment of GIST48B cells for 24 h with nutlin-3 lead to a relative reduction of MDM2 binding to p53 (Fig. 3C). RITA treatment did not seem to affect the binding of MDM2 to p53 (Fig. 3C).

To further elucidate the mechanisms of action of MDM2i, we also examined the binding of heterogeneous nuclear ribonucleoprotein K (hnRNP K), a p53 cofactor, to MDM2 before and after treatment. Neither nutlin-3 nor RITA affected the overall level of hnRNP K expression in our western blot analysis (Fig. 3A/B/C). However, immunoprecipitation showed that the binding between MDM2 and hnRNP K was significantly reduced after nutlin-3 treatment but not after RITA treatment (Fig. 3C). This could indicate that after RITA treatment MDM2 can still bind and potentially degradate hnRNP K.

### Phosphorylation of p53 at Serine 46

Several kinases are able to activate p53 by phosphorylation at different sites. Phosphorylation of Serine 46 is known to initiate apoptosis, rather than induce cell cycle arrest. One of the kinases known to phosphorylate at that site is the homeodomain-interacting protein kinase 2 (HIPK2) [14]. Rinaldo et al. have proposed that nutlin-3 treatment degrades and RITA treatment activates HIPK2, leading to a stronger phosphorylation of p53 and subsequently to a stronger induction of apoptosis [15]. We therefore performed p53 and phospho (Serine 46)-p53 immunoprecipitations to analyse whether nutlin-3 or RITA treatment has any effects on p53 phosphorylation at Serine 46.

However, the phospho p53 and total p53 staining revealed that both nutlin-3 and RITA induced phosphorylation at Serine 46 (Fig. 3C).

To address the total levels of HIPK2 in our cell lines before and after treatment with MDM2i, we performed another western blot analysis. In GIST48B treatment with RITA and nutlin-3 did not alter the level of HIPK2 expression (Fig. 3D). However, in other cell lines the baseline expression of HIPK2 was lower and RITA treatment led to a downregulation of HIPK2, as shown for GIST430 (Fig. 3D).

### Nutlin-3 enhances the induction of apoptosis of different KIT-inhibitory drugs

We also studied the effects of combinational treatment of nutlin-3 and different direct and indirect KIT-inhibitory drugs using therapeutically relevant doses of nutlin-3 [11].

**Figure 4:**
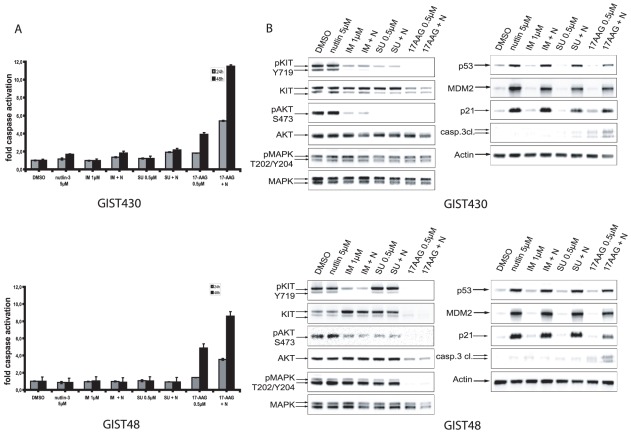
Analysis of nutlin-3 in combinational treatment. A: Induction of apoptosis represented by amount of activated caspases 3 and 7 in GIST430 and GIST48. Indicated concentrations of nutlin-3 were assayed alone and in combination with different KIT-inhibitors after 24 h and 48 h of incubation. DMSO was vehicle control. Bars, mean of quadruplicate cultures with SD represent multitudes of DMSO-only values. B: Western Blot analyses after 24 h of incubation with doses of nutlin-3 (5 µM) alone and in combination with different KIT-inhibitors in GIST430 and GIST48. N = nutin-3, IM = imatinib mesylate, SU = sunitinib.

Adding nutlin-3 to IM in IM-resistant GIST430 and GIST48 had little or no effect on caspase 3 and 7 activation (Fig. 4A). The HSP90-inhibitor 17-AAG potently inhibits oncogenic KIT regardless of secondary IM-resistance mutations. A combination of 17-AAG and nutlin-3 resulted in an additive proapoptotic effect in the IM-resistant cell lines GIST430, GIST48 and GIST48B, as measured by western blot-, luminescence- and annexin V/7-AAD-assays, shown for GIST430 and GIST48 (Fig. 4A/B/FigS3).

### Nutlin-3 has no effect on KIT-dependent signaling pathways

Constitutively activated KIT represents the key oncogenic driver in GIST that promotes proliferation and has antiapoptotic effects. Thus, any disruption within the KIT-signaling pathway may directly influence the growth and survival of cells. To exclude possibly antagonistic off-target effects within this signaling pathway we treated cells with nutlin-3 alone and in combination with different KIT-inhibitory drugs. Western blot analyses were performed to investigate the effects of nutlin-3 treatment on KIT-signaling in the presence or absence of KIT-inhibitory drugs.

IM treatment alone only partially inhibited phosphorylation of KIT and AKT, yet not of MAPK in both GIST430 and GIST48 (Fig. 4B), as typically observed in imatinib-resistant cells. Sunitinib (SU) treatment alone inhibited phosphorylation of KIT and AKT in GIST430, but not in GIST48 (Fig. 4B), which is expected as SU potently inhibits secondary KIT exon 13 mutations (GIST430) but not kinase loop mutations in exon 17 (GIST48). The HSP90 inhibitor 17-AAG, which has been shown to inhibit KIT regardless of secondary mutations, inhibits KIT and KIT-dependent signaling in both cell lines. IM, SU and 17-AAG did not affect expression levels of p53, MDM2 or p21 at this time point (Fig. 4B).

Nutlin-3 treatment did not affect expression levels of KIT and KIT-dependent signaling pathways in any combination (Fig. 4B). In addition, no antagonistic effects were seen in any combination, including imatinib, sunitinib, sorafenib, 17-AAG, SAHA, nilotinib and dasatinib (data not shown), as measured by SRB assays in any tested GIST cell line.

### P53 transcriptional levels are low in GIST-T1 and GIST882

**Figure 5:**
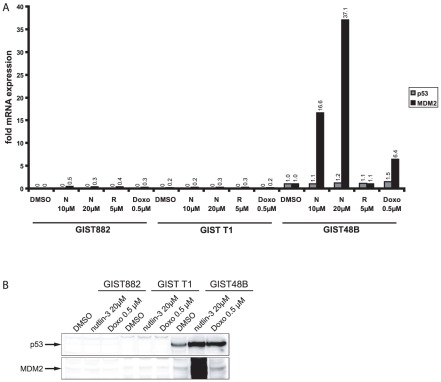
Effects of different inhibitors on p53 and MDM2 mRNA and protein levels. A: Quantitative Real Time RT-PCR evaluation of p53 and MDM2 mRNA in GIST882, GIST T1 and GIST48B after treatment of different concentrations of nutlin-3 (10 µM and 20 µM) and RITA (5 µM). Values were normalized to the DMSO value for each point in time. B: Western Blot studies of GIST882, GIST-T1 and GIST48B treated with nutlin-3 20 µM and Doxorubicin 0.5 µM for 24 h. N = nutlin-3, Doxo = Doxorubicin.

To gain further understanding of the differential sensitivity of GIST cells to nutlin-3, we performed a quantitative real time PCR evaluation of *TP53* and *MDM2* mRNA in GIST882, GIST-T1 and GIST48B. In GIST48B nutlin-3 and RITA treatment resulted in a small (10%) and treatment with doxorubicin resulted in a stronger increase (50%) in *TP53* mRNA levels (Fig. 5A). *MDM2* mRNA levels were strongly increased by nutlin-3 (16- to 37-fold) and doxorubicin treatment (6-fold), but only mildly after RITA treatment (10%) (Fig. 5A). In GIST882 and GIST-T1 however, *TP53* mRNA levels were not detectable (Fig. 5A). *MDM2* mRNA levels were slightly increased by nutlin-3- and doxorubicin treatment and minimally reduced after RITA treatment (Fig. 5A). These findings correlate with our findings in the western blot analysis: In contrast to GIST48B p53 and MDM2 proteins were not detectable in GIST882 and GIST-T1 despite treatment with high doses of nutlin-3 or doxorubicin (Fig. 5B).

### Analysis of the p53 homologes p63 and p73 and p53 dependent proteins

It has been shown that nutlin-3 treatment can induce apoptosis in higher doses in a p53 independent manner, via p73 [12]. Therefore, we analyzed the expression of different members of the p53 family, p63 and p73, in response to MDM2i treatment in different cell lines. P73 expression levels were high in all the GIST cell lines (FigS4A) compared to the positive control cell line MCF7 and HeLa. P73 expression was slightly increased (2-fold) after nutlin-3 treatment in GIST882 and GIST430 but not in the other cell lines (FigS4A). P63 was undetectable in GIST430, GIST48 and GIST-T1 and weakly expressed in GIST882 and GIST48B.

The proapoptotic protein BAX, known to be transcriptionally regulated by p53, was expressed in all the GIST cell lines, with higher levels in p53 positive cell lines (2–3 -fold) (FigS4A). In these cell lines, nutlin-3 also mildly induced BAX while RITA treatment had no effect on BAX levels (FigS4A).

## Discussion

Complete pathological remissions of metastatic GISTs following treatment with IM represent a rare exception. Mechanisms of (late) resistance to IM have been widely described and comprise secondary mutations with a large degree of genomic heterogeneity [16], loss of c-KIT expression [5], *c-kit* amplification, and rarely BRAF mutations [17]. It is of yet unknown, whether these changes occur upon treatment with imatinib or if clones with pre-existing secondary mutations are selected for during treatment.

Treatment of imatinib-sensitive cell lines with IM in vitro causes only modest induction of apoptosis [18] suggesting, that combinational treatments with proapoptotic drugs may be needed to push cells into apoptotic death. For proof of concept studies we used two p53 modulating drugs, nutlin-3 and RITA, which have been intensively studied in the past years. Nutlin-3 is a cis-imidazoline analogue that binds MDM2 in the p53 binding pocket and thus inhibits interaction with p53. Subsequently MDM2 can no longer repress p53 by blocking its transactivation site and by tagging it for proteasomal degradation [11]. RITA is a furanic compound, which is thought to bind, among other molecules, to the p53 NH2-terminal domain, inhibiting it from binding to MDM2. This is supposed to preserve MDM2 E3-ligase activity and MDM2 mediated degradation of other MDM2 clients. It has been proposed that some of these other clients might be anti-apoptotic p53 cofactors and by preserving MDM2 E3-ligase activity the apoptotic response to p53 activation might be stronger after RITA treatment, compared to the nutlin-3 treatment [15] [19].

Our studies showed a surprisingly differential sensitivity of the panel of GIST cell lines towards nutlin-3 and RITA. Nutlin-3 treatment alone showed only moderate growth inhibition and weak induction of apoptosis in IM-resistant cell lines, but no effects in the IM-sensible GIST882 and GIST-T1 (Fig. 1). This corresponded well with the observation that, in contrast to IM-resistant GIST cell lines, GIST882 and GIST-T1 neither had baseline expression of p53 nor showed induction of p53 upon treatment with high doses of nutlin-3 or doxorubicin (Fig. 3, Fig. 5B). Since the lack of nutlin-3 response is predictive of dysfunctional p53 we performed quantitative RT-PCR for p53 and MDM2 following nutlin-3, RITA and doxorubicin treatment, which did not result in a detectable induction of p53 in GIST882 and GIST-T1. We were unable to amplify the promotor region in GIST882 and exon 2 through 7 in GIST-T1 (Fig. 5C) suggesting a homozygous deletion. A large deletion was excluded by 250K SNP arrays in both cell lines (J. Fletcher, data not shown). This finding is noteworthy given the wide use of both cell lines for drug validation studies in GIST and the potential bias this may cause for studying apoptosis in these cell lines. Very little data is available on the incidence of p53 mutations in GIST. A study by Romeo et al. in patients with untreated metastatic GISTs (n = 353) found 16% of mutations (n = 13) in a subgroup of patients with high p53 and low p21 expression (n = 79) [13]. We performed a mutational analysis in 62 additional tumors of both localized and metastatic GISTs regardless of p53 expression data (Tab. 1 and Tab. 2). While the P72R polymorphism was commonly found in the majority of our patients, classical *TP53* mutations were rare (3%). Our data therefore support the previous data, that *TP53* mutations in GIST may be an exception at least in untreated patients, which would be a prerequisite for the clinical use of p53-modulating drugs in GIST. While other genetic events have been described to dysregulate the p53 pathway in GIST, such as deletions of p16 or amplification of MDM2, these would not preclude the use of MDM2 inhibitors but could rather sensitize cells to a treatment [20], [21]. To our surprise, two out of the five commonly used GIST cell lines harbor deletions in *TP53*. Cultivation of GIST cells is particularly difficult and most short-term cultures exhibit senescence after few passages. However, *TP53* mutations may represent a selection advantage in vitro, which might explain the presence in cell lines that were derived from imatinib-naïve metastatic GISTs. Although the P72R polymorphism is very commonly found in the healthy population it has nonetheless been associated with familiar breast cancer in Jewish women. In our study, 60% of GIST patients had a homozygous polymorphism compared to 22% in the historical control [22].

Interestingly, p53 induction by nutlin-3 resulted in a consistent antiproliferative effect in p53 wild-type GIST but not in a strong induction of apoptosis. As hypothesized by Enge et al, this may be due to the induction of the anti-apoptotic protein p21, which was seen after nutlin-3 but not after RITA treatment (Fig. 3) [19]. Our findings suggest that in the GIST context, when given alone, nutlin-3 induces cell cycle arrest rather than apoptosis (FigS2). This may be a tumor specific effect, as Vasilev and colleagues recently showed that p21 may not decrease the apoptotic response to nutlin-3 in other cell systems [23]. Of note, GIST cell lines showed different sensitivity profiles towards RITA and nutlin-3. The group of Selivanova has recently described several mechanisms that may explain differential effects seen with RITA and nutlin-3 involving the p53 co-factor hnRNP K as well as the p53-activator HIPK2 [15], [19]. HnRNP K has been described by Enge et al. as an important cofactor of p53 induced cell cycle arrest [19]. HnRNP K is also a client of MDM2 and it has been proposed that RITA, in contrast to MDM2-bound nutlin-3, causes degradation of hnRNP K by the fully functional MDM2. Enge et al. showed that RITA treatment decreased both the overall levels of hnRNP K and the binding of hnRNP K to MDM2 [19]. Selivanovas group also showed that nutlin-3 treatment downregulated HIPK2 while RITA treatment resulted in kinase activation. The subsequent phosphorylation of p53 at Serine 46 was reported to contribute to the rather proapoptotic effects of RITA [15]. In GIST, RITA did not have an effect on hnRNP K expression. Regarding HIPK2 activation, RITA treatment did not induce a stronger phosphorylation of p53 at S46, compared to the nutlin-3 treatment. All this implicates a different mechanism of action for RITA in this cellular context.

While p53 mutational status was predictive for response to nutlin-3, RITA showed a differential activity in the same GIST cell line panel. GIST48B and GIST-T1 were particularly sensitive to RITA, despite the absence of p53 in the latter. Notably, these effects were not a result of an activation of p63 or p73, which has previously been described as an alternative mechanism for induction of apoptosis by MDM2-inhibitory drugs [12]. In addition, in contrast to nutlin-3, RITA also did not inhibit the MDM2/p53 interaction as shown by immunoprecipitation in GIST48B (Fig. 3C). Supporting evidence for p53-independent effects of RITA were also found in the more widely used osteosarcoma cell line SAOS-2 [24] (p53-null) (FigS4C). A potential explanation for p53-independent effects of RITA were reported by Yang et al, who described a novel mechanism of action for RITA through induction of DNA-damage response [25]. Proapoptotic function of mutated p53 may also be rescued by RITA as shown by Zhao et al. [26]. Together with our findings, these data suggest a more complex and less predictable mechanism of action for RITA.

Future studies should therefore investigate the early events preceding the onset of apoptosis in GIST-T1 and GIST48B following RITA treatment, which may help to identify additional targets for proapoptotic treatments.

Combinational treatments with several KIT-inhibitory drugs and the HSP90 inhibitor 17-AAG showed additive proapoptotic effects with nutlin-3 if effective KIT-inhibition was achieved. KIT and KIT-depending signaling pathways were not affected by nutlin-3 alone, nor did nutlin-3 influence the effect of KIT-inhibitory drugs on KIT-dependent signaling. In GIST430, a cell line harboring an IM-resistance mutation in exon 13, nutlin-3 treatment improved the apoptotic response to sunitinib, which is known to effectively inhibit KIT exon 13 mutations [27]. In contrast, GIST48, harboring an exon 17 resistance mutation, nutlin-3 did not show additive effects with imatinib or sunitinib. However, a pronounced additive effect was seen with 17-AAG, which effectively inhibits KIT-activation regardless of underlying KIT mutations [28]. These results imply that a combinational treatment with p53-activating drugs would require an effective shut down of the oncogenic KIT-signal. This correlates with the results of Kurosu et al., who were able to show that nutlin-3 treatment enhanced the induction of apoptosis of IM treatment in BCR/ABL positive leukemia cell lines [29]. Importantly, no antagonistic effects have been observed with any treatment combination.

In summary, our studies provide the first promising evidence that modulators of the MDM2/p53 pathway may enhance the apoptotic response to KIT-inhibitory treatments in GIST. In the GIST context, nutlin-3-type inhibitors seem more predictable than furanic compounds since their activity relies on functional p53.

## Materials and Methods

### Cell lines

GIST-T1 was established from a human, untreated, metastatic GIST containing a 57 bp deletion in *KIT* exon 11 which is highly sensitive to IM at low nanomolar doses [30]. GIST-T1 harbours a homozygous deletion. GIST882, as previously described, was established from an untreated human GIST with a homozygous missense mutation in *KIT* exon 13, encoding a K642E mutant KIT oncoprotein which is sensitive to IM [31]. We were unable to amplify the *TP53* promotor region, also suggesting a homozygous deletion in this region. Both GIST-T1 and GIST882 were p53 negative as measured by qRT-PCR and western blot analysis. GIST48 was established from a GIST that had progressed, after initial clinical response, during IM therapy. GIST48 has a primary, homozygous *KIT* exon 11 missense mutation (V560D) and a heterozygous secondary *KIT* exon 17 (kinase activation loop) mutation (D820A) [28]. GIST48 is IM-resistant, due to a secondary *KIT* exon 17 mutation. GIST48B is a subline of GIST48 which, despite retaining the activating *KIT* mutation in all cells, expresses KIT transcript (data not shown) and protein at essentially undetectable levels [32]. GIST430 was also established from a GIST that progressed during IM therapy. It has a primary heterozygous *KIT* exon 11 in-frame deletion and a heterozygous secondary *KIT* exon 13 missense mutation that confers IM resistance [28]. GIST48, GIST48B and GIST430 are *TP53* wild type and express p53 RNA and protein as measured by qRT-PCR and western blot analysis. The liposarcoma cell line LPS141 is highly sensitive to MDM2 inhibition due to high-level amplification of MDM2 and served as positive control [32]. All cell lines except for GIST-T1 have been established at the Brigham and Women's Hospital in Boston, USA, by the Group of Professor Jonathan A. Fletcher. GIST-T1 has been established by Takahiro Taguchi at the Kochi University in Japan.

### Reagents and Antibodies

Nutlin-3 (racemate of nutlin-3a and its inactive enantiomere nutlin-3b) was purchased from Sigma Aldrich. RITA and SAHA were purchased from Cayman Chemical (Ann Arbor, Michigan). Imatinib (IM) and sunitinib (SU) were purchased from Sequoia Research Products (Pangbourne, UK). Dasatinib, Nilotinib and Sorafenib were purchased from LC Laboratories (Woburn, MA). 17-N-Allylamino-17-demethoxygeldanamycin (17-AAG) was purchased from Calbiochem (Merck, Darmstadt, Germany).

A rabbit polyclonal antibody to KIT was from DAKO (Carpinteria, CA). Polyclonal rabbit antibodies to phospho-KIT Y703 and mouse MDM2 antibody were from Zymed Laboratories (South San Francisco, CA). Polyclonal rabbit antibodies to phospho p53, p53, total p42/44 mitogen-activated protein kinase (MAPK), phospho-p44/42 MAPK T202/Y204, phospho-AKT S473, total AKT, phospho-KIT Y719, cleaved caspase-3 were from Cell Signaling (Beverly, MA). p21 and Beta Actin antibodies were purchased from Sigma (St. Louis, MO). Mouse p53 antibody was purchased from BD Pharmingen (Franklin Lakes, NJ), mouse hnRNP K antibody from Imuno Quest (North Yorkshire, UK) and MDM4 rabbit antibody from Bethyl Laboratories (Montgomery, TX). HIPK2 antibody was kindly provided by Prof Lienhard Schmitz (University of Giessen).

### In vitro assays

Viability studies were carried out using a Sulforhodamin (SRB) assay [33]. For these studies, the cell lines were plated at 15,000 to 30,000 cells per well in a 96-well flat-bottom plate (Falcon, Lincoln, NJ), cultured in serum-containing media for 1 day, and then incubated for 72 or 144 hours with MDM2-, or KIT-inhibitors and DMSO-only solvent control. The SRB assay absorption was measured with a Genion Luminometer (Tecan, Crailsheim, Germany) and the data were normalized to the DMSO-only control group. All experimental points were measured in triplicate or quadruplicate wells for each plate and were replicated in at least two plates.

Apoptosis studies were done by measuring caspase-3 and caspase-7 activation with the Caspase-Glo 3/7 Assay Kit (Promega). This assay uses a proluminescent substrate containing the DEVD sequence recognized and activated by caspase-3 and caspase-7 [34], [35] and the luminescence signal is proportional to net caspase-3 and caspase-7 activation [36]. The experimental conditions were all as described above for the SRB studies except treatment duration of 24 and 48 hours.

### Western blotting and immunoprecipitation

Protein lysates were prepared from cell line monolayers according to standard protocols [37]. Protein concentrations were determined with the Bio-Rad Protein Assay (Bio-Rad Laboratories, Hercules, CA). Electrophoresis and immunoblotting were carried out as previously described [38]. Changes in protein expression and phosphorylation as visualized by chemiluminescence were captured and quantified using a FUJI LAS3000 system with Science Lab 2001 ImageGauge 4.0 software (Fujifilm Medial Systems, Stamford CT, USA).

Immunoprecipitations were performed with Sepharose protein G beads (Zymed Laboratories) from 400–600 µg of total protein as described previously [34].

### Cell cycle analysis

Cells were plated in six-well plates, grown until 80% confluence, and then treated for 48 hours with DMSO, nutlin-3 (5 µM and 10 µM), RITA (1 µM and 5 µM) or IM (500 nM). Cells were then trypsinized and stained with DNA prep containing propidium iodide (PI) (Beckman Dickonson, Heidelberg, Germany) followed immediately by flow cytometric analysis (BeckmanCoulter FC500 Flow Cytometer). Modfit LT software 3.1 (Verity Software House, Topsham, ME) was used for data analysis.

### Cell viability assessment by Annexin V Staining

After drug treatments as described for the cell cycle analysis above, cells were resuspended in 500 µl of the staining buffer and annexin V FITC (525 nm) and 7-AAD (675 nm) (BD) were added. After incubation at room temperature for 15 min, annexin V-positive cells were estimated by flow cytometry. 10000 events of each sample were acquired on a BeckmanCoulter FC500 Flow Cytometer. Doublet discrimination was done with FL2 vs. FL2 peak histogram.

### Quantitative Real Time Reverse Transcriptase-PCR

Cells were plated in 12-well plates, grown until 80% confluence, and then treated with drug-containing media. Cells were then collected with RNAprotect Cell reagent (Qiagen, Hilden, Germany) and processed with RNeasy Mini Kit (Qiagen) according to the manufacturer's protocol, for RNA isolation. cDNA was transcribed by Reverse Transcriptase-PCR using random primers and KIT-cDNA was amplified and measured using TaqMan gene expression assays (Applied Biosystems, Softer City, CA) and Real Time PCR system LightCycler 480 (Roche, Grenzach-Wyhlen, Germany). Beta-Actin cDNA, (also measured using TaqMan gene expression assay (AB)) served as reference gene for relative quantification.

### P53 sequencing of GIST cell lines and a panel of primary GIST

Genomic DNA was extracted from 10 µm sections of the paraffin-embedded tumor blocks used for immunohistochemistry, using a macrodissection technique to reduce contamination with nonneoplastic tissue, from fresh frozen tumor samples and from cell lines. Genomic DNA was isolated using the QIAmp DNA Mini Kit. PCR was done on *TP53* for exons 4 to 9 (primers are listed in Tab. 4) in GIST tumors and for exons 1 to 12 in all GIST cell lines. 10 µL PCR reactions consisted of 20 ng input genomic DNA, 10× HotMAster Taq buffer with 25 mM MgCl2, 2.5 units HotMaster Taq DNA polymerase (5 Prime). PCR was carried out at an initial denaturation step of 2 min at 95°C, followed by 40 cycles of 20 s at 95°C, 10 s at 60°C, and 35 s at 65°C, and a final elongation step of 10 min at 72°C. Nucleotide sequence analysis was performed with the BigDye Terminator v1.1 Cycle Sequencing Kit (Applied Biosystems, Foster City, CA, USA). The purified PCR products were sequenced in both directions. Cycle sequencing products were analyzed using the ABI PRISM 310 Genetic Analyser (Applied Biosystems, Darmstadt, Germany).

**Table 4:** Primers used for the *TP53* Sequencing.

Exon	Forward Primer	Reverse Primer
Promotor	5′-TCACAGCTCTGGCTTGCAGA-3′	5′-CCACTCACCCCCAAACTCGC-3′
1	5′-GTTGATGGGATTGGGGTT-3′	5′-CAGTCAGGAGCTTACCCAAT-3′
2–3	5′-ATCCCCACTTTTCCTCTTGC-3′	5′-GAAAAGAGCAGTCAGAGGAC-3′
4	5′-CGTTCTGGTAAGGACAAGGG-3′	5′-ACAAAAGAAATGCAGGGGG-3′
5–6	5′-TCTTTGCTGCCGTCTTCC-3′	5′-AGGGCCACTGACAACCAC-3′
7	5′-TGCTTGCCACAGGTCTCC-3′	5′-GTCAGAGGCAAGCAGAGGC-3′
8–9	5′-GGACAGGTAGGACCTGATTTCC-3′	5′-AAACAGTCAAGAAGAAAACGGC-3′
11	5′-TTACTTCTCCCCCTCCTCTGTTG-3′	5′-GCTTTCCAACCTAGGAAGGCAG-3′
12	5′-TCATCTCTCCTCCCTGCTTCTGTC-3′	5′-TGCTTCTGACGCACACCTATTG-3′

### Whole transcriptome sequencing

rRNA was depleted from 5 µg of total RNA using biotinylated oligonucleotides (Ribominus, Invitrogen), and libraries were constructed from the rRNA-depleted RNA according to the SOLiD Total RNA-seq Kit Protocol (Applied Biosystems). Briefly, library construction involved fragmentation of the RNA by RNAse III to an average size of 150 bases, ligation of the fragmented RNA to adaptors in a directed orientation, then cDNA synthesis and PCR amplification of the resulting library. Approximately 50 bases were sequenced from one end of each fragment using either the SOLiD 3+ or SOLiD 4 instrument and reagents (Applied Biosystems). The resulting sequence data was mapped to the human reference genome, hg18, using Bioscope v1.2 (Applied Biosystems). Sequences that mapped to unique locations were quantified per transcriptional unit, as defined in RefSeq (release 35), as “reads per kilobase of transcript per megabase of total sequence (RPKM).”

A weighted score, described by the following expression:
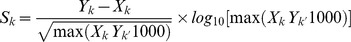
was used to rank the difference in normalized reads for each transcript between samples.

## Supporting Information

Figure S1 Whole transcriptome sequencing data for p53 demonstrates no detectable levels of p53 in GIST882 compared to regular transcriptional levels in GIST430 and GIST48. N = nutlin-3.
(EPS)

Figure S2 Cell cycle analysis FACS assay in GIST48 (A), GIST48B (B) and GIST T1 (C). Inhibitor concentrations were used as indicated and assayed after 48 h of incubation. DMSO was used as vehicle control. N = nutin-3, IM = imatinib mesylate, SU = sunitinib.
(EPS)

Figure S3 Induction of apoptosis as measured by an Annexin/7AAD based FACS Assay in GIST430 (A) and GIST48 (B). N = nutin-3, IM = imatinib mesylate.
(EPS)

Figure S4 Analysis of apoptosis related proteins and effects of MDM2i in p53-null SAOS-2 cells. A: Western Blot analyses after 24 h of incubation with doses of nutlin-3 (5 µM) and RITA (1 µM) in GIST48, GIST48B, GIST-T1, GIST882 and GIST430. HELA and MCF-7 cell serve as positive controls for p63 and p73. B: Sulforhodamin-based cytotoxicity assays for nutlin-3 and RITA in p53-null SAOS-2 cell line. C: Induction of apoptosis represented by amount of activated caspases 3 and 7 in SAOS-2. Indicated concentrations of the inhibitors were assayed after 24 h and 48 h of incubation. DMSO was vehicle control. Bars, mean of quadruplicate cultures with SD represent multitudes of DMSO-only values.
(EPS)
